# Effects of traditional Chinese medicine S*hu Gan Jian Pi* granules on patients with breast cancer and cancer-related fatigue: study protocol for a randomized controlled trial

**DOI:** 10.1186/s13063-015-0723-0

**Published:** 2015-04-26

**Authors:** Chen Li, GuoWang Yang, MingWei Yu, YongMei Xu, Na Xue, Nan Nan, XiaoMin Wang

**Affiliations:** Oncology Department, Beijing Hospital of Traditional Chinese Medicine affiliated with Capital Medical University, No. 23, Back Road of Art Gallery, Beijing, Dong Cheng District 10010 China

**Keywords:** Traditional Chinese Medicine, Breast cancer, Cancer-related fatigue

## Abstract

**Background:**

Cancer-related fatigue (CRF) is a common and often long-lasting symptom for many breast cancer survivors. Evidence for its management is scarce. However, the Traditional Chinese Medicine (TCM) *Shu Gan Jian Pi* (SGJP) granules is an effective and practical therapy for CRF.

**Methods/Design:**

We will conduct a multicenter, randomized, double-blind, placebo-controlled clinical trial to determine whether the SGJP granules can effectively manage CRF. Breast cancer survivors experiencing fatigue within 5 years of primary treatment completion will be enrolled and randomly assigned to Group S (SGJP) or Group P (placebo). The primary outcome measures will include Revised Piper Fatigue Scale score. Outcome measures will be collected at baseline and at weeks 2, 4, and 8.

**Discussion:**

This study’s findings may contribute to the development of an effective intervention for CRF.

**Trial registration:**

Current controlled trials ISRCTN12702489, 14 August, 2013.

## Background

Cancer-related fatigue (CRF) has been documented as one of the most distressing symptoms of breast cancer survivors [1]. It is defined as a distressing, persistent, subjective sense of physical, emotional, and/or cognitive feeling of tiredness or exhaustion related to cancer or cancer treatment that is not proportional to recent activity and interferes with usual functioning [2]. Prevalence rates for CRF of breast cancer are reportedly 58 to 94% during treatment and between 56% and 95% after adjuvant chemotherapy [1]. Cancer survivors frequently report that they never regain their pre-diagnostic energy level, which diminishes their quality of life [3]. Many common problems experienced by breast cancer survivors may be associated with CRF [4]. Problems associated with fatigue may be the result of the cancer itself, its treatments, and/or other comorbid conditions [5,6]. The pathogenesis of CRF has not been thoroughly described and several mechanisms can contribute to its development. Non-pharmacological measures that have shown promise include education, cognitive-behavioral therapy, exercise, and sleep therapy [1,7]. Pharmacological measures that have shown potential include the psycho-stimulants methylphenidate, dexmethylphenidate, erythropoietin, darbepoetin, progestational steroids (medroxyprogesterone acetate, megestrol acetate), modafinil, and paroxetine [1,7]. However, only small differences were noted between pharmacological treatments and placebo.

Traditional Chinese Medicine (TCM) is one of the most common interventions used in China. *Shu Gan Jian Pi* (SGJP) was changed from the classical formula *Xiao Yao San* that was derived from *Taiping Huimin Heji Ju Fang*, which was originally compiled by the government of the Han dynasty. CRF belongs to the syndrome of liver *qi* stagnation and splenic *qi* asthenia in TCM rationale. The liver possesses the physiological functions of dredging and regulating. The failure of the liver to disperse the stagnation of *qi* due to emotional depression results in breast cancer patients manifesting emotional depression and migratory pain in the chest. If the liver fails to dredge and disperse,the liver will invades the spleen, it will cause dysfunction of the muscles of the four limbs. Thus, breast cancer patients manifest fatigue.

SGJP granules can relieve the syndrome; SGJP is composed of six herbs including *Radix astragali* (*Huang Qi*), *Radix bupleuri* (*Chai Hu*), *Radix angelicae alba* (*Dang Gui*), and three other herbs. The action of *Radix astragali* (*Huang Qi*) is to replenish *qi*. That of *Radix bupleuri* (*Chai Hu*) is to disperse the stagnated liver-*qi*. Four other herbs have the same actions. Many patients in China turn to TCM with the complaint of fatigue after cancer treatment. One study reported that TCM demonstrated the ability to relieve CRF [8]. We have attempted to treat CRF in clinical practice without success. Although SGJP granules have demonstrated positive effects on relieving CRF, rigid validation using a randomized controlled trial remains the best way to examine the effects of SGJP in patients.

## Methods/Design

### Study design

**Figure 1:**
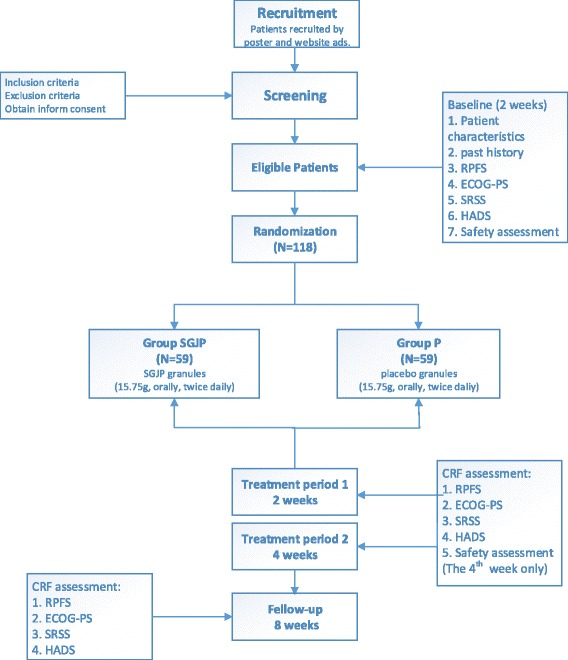
Flow of patients through the trial. 1. RPFS: Revised Piper Fatigue Scale. 2. ECOG-PS: Eastern Cooperative Oncology Group Performance Status. 3. SRSS: Self-Rating Scale of Sleep. 4. HADS: Hospital Anxiety and Depression Scale. 5. Safety assessment: routine blood test, routine feces test, routine urine test, liver and kidney function tests and electrocardiogram.

This study will use a two-group multicenter, randomized, double-blind, and placebo-controlled clinical trial design in which participants will receive either enhanced TCM or placebo. We will enroll 118 patients with breast cancer. Participants will be recruited from three centers: Beijing Hospital of Traditional Chinese Medicine, Beijing Cancer Hospital, and People’s Hospital of Beijing Da Xing District. The study schema and estimated recruitment numbers are presented in Figure 1. Randomization will be used to allocate patients to groups subjected to a 4-week treatment period and a 4-week follow-up period. Four visits will be scheduled for each patient: baseline and weeks 2, 4, and 8.

### Ethical issues

The study received ethical approval from the Research Ethical Committee of Beijing Hospital of Traditional Chinese Medicine Affiliated with Capital Medical University (number 201338) and registered with the International Standard Randomized Controlled Trial Number Register (ISRCTN 12702489). Patients willing to participate will sign a consent form prior to participating.

### Setting and patients

The study will include breast cancer patients with the complaint of moderate to severe fatigue. Patients will be screened for fatigue using the Revised Piper Fatigue Scale (RPFS) to identify those with significant fatigue. Table 1 will show the detailed eligibility and exclusion criteria. Recruitment will occur at three centers in Beijing: Beijing Hospital of Traditional Chinese Medicine, Beijing Cancer Hospital, and People’s Hospital of Beijing Da Xing District.

**Table 1:** **Eligibility criteria for the randomized clinical trial**

Inclusion criteria	
1	Patients have definite outpatient pathologic diagnosis of breast cancer
2	Eligible patients had completed chemotherapy and/or radiotherapy at least 1 month and mastectomy within 5 years
3	Stage I to III breast cancer with no evidence of recurrence and metastasis
4	Eastern Cooperative Oncology Group Performance Status 0 to 2
5	Traditional Chinese Medicine (TCM) syndrome is differentiated as liver depression and spleen deficiency
6	Anticipated survival time exceeds 6 months
7	Eligible patients have no plan to receive chemotherapy and/or radiotherapy during the study
8	Revised Piper Fatigue Scale score ≥ 4
9	Provided signed informed consent before enrollment
Exclusion criteria	
1	Complicated by serious diseases of heart, liver and kidney, immune and hematopoietic systems
2	Children and pregnancy
3	Receiving active treatment for anemia with erythropoietin or blood transfusions
4	Using steroids to cure cancer-related fatigue
5	Diagnosed with depression, mental disease or cognitive impairment
6	Allergic to a Chinese herbal compound

### Intervention

If patients meet the inclusion criteria, they will take a randomly generated number to the pharmacy and the pharmacist will give them SGJP or placebo in a package containing either 15.75 mg medication or dextrin (90%) and *Herba pogostemonis* (10%). Patients will take one package twice daily. The intervention period will be over 4 weeks. After 4 weeks on the prescribed medication, the patients will stop taking SGJP or placebo for another 4 weeks and then undergo the clinical evaluation.

The intervention group treatment is composed of six herbs including *Radix astragali* (*Huang Qi*), *Radix bupleuri* (*Chai Hu*), *Radix angelicae alba* (*Dang Gui*), and three other herbs. The ingredients of SGJP cannot be disclosed because the formula is currently being patented. The control group formulation includes dextrin (90%) and *Herba pogostemonis* (10%). The study medicine was made by Beijing Tcmages Pharmaceutical Co., LTD, Beijing, China. Based on the infrared fingerprint spectrum techniques and Good Manufacturing Practice**s** (GMP) full range management, this company can guarantee the consistency of granule composition.

### Assessment

#### Primary outcome measure

*Revised Piper Fatigue Scale* (RPFS), a multidimensional assessment tool that subjectively measures the level of fatigue of patients with cancer, has been widely used in research. It consists of 22 items, each rated on a visual analog scale with a score ranging from 0 (least) to 10 (most). The items are divided into four dimensions: severity, affective meaning, sensory, and cognition [9].

#### Secondary outcome measure

*Eastern Cooperative Oncology Group Performance Status* (ECOG-PS) is a scale to evaluate performance status. It is based on five levels [10].

*The Self-Rating Scale of Sleep* (SRSS) is a frequently-used instrument in China that consists of 10 items that measure sleep quality. Each item is rated on a scale of 1 (best status) to 5 (worst status) [11].

*The Hospital Anxiety and Depression Scale* (HADS) was developed to identify anxiety disorders and depression among patients. It is divided into an Anxiety subscale and a Depression subscale, both of which contain seven intermingled items. Each item is rated on a scale of 0 (best status) to 3 (worst status) [9].

#### Safety assessments

To assess the safety of SGJP, we will perform the following tests on participants at the screening phase (baseline) and after treatment (week 8): routine blood, routine feces, routine urine, liver and kidney function, and electrocardiography.

#### Sample size

Based on our previous study, we have baseline data from Chinese breast cancer patients with CRF in Beijing, China [12]. Although the study was published in a Chinese journal, we considered it is the most factual data. RPFS was the primary outcome in our previous study. The planned sample size is 118 patients with an anticipated 15% attrition rate. With this sample size, there will be in excess of a 95% power and a (1-side) 5% significance level in detecting treatment differences. The standard deviation is 1.27. The study was planned to detect a one-unit difference in change scores between the two groups.

#### Randomization and blinding

Eligible patients will be randomized into the groups SGJP and placebo (P) in a 1:1 ratio for a target total of 118 patients. Patients will be randomized in blocks of four by **Statistical Analysis System** (SAS). This will be a double-blind study in which the participants and investigators are blinded. The patients will receive granules either with SGJP or placebo. These granules have the same taste, appearance, and color.

#### Analysis

Statistical analyses will be performed by the Statistical Package for Social Science statistics (SPSS 18.0). Continuous variables will be expressed as median and standard deviations. Groups will be compared using the *t*-test or Student’s *t*-test, as appropriate, based on the data distribution. Categorical variables will be expressed as percentages. Groups will be compared using the chi-square or Fisher’s exact test, as appropriate, based on the expected counts. Patient characteristics and past history will be reported and compared between groups. Descriptive statistics will be presented to describe the trial results. Student’s *t*-test will be applied to fatigue change scores at every time point between the groups. Similar analyses will be performed for other outcomes (ECOG-PS, SRSS, HADS). Values of *P* < 0.05 will be considered statistically significant.

#### Data collection

We will collect basic data from each patient including their characteristics and past history of cancer, outcomes, and safety assessment data at baseline and follow them up at 2, 4, and 8 weeks (Table 2).

**Table 2:** **Study visits**

	**Visit 1**	**Visit 2**	**Visit 3**	**Follow-up**
	**Baseline**	**Week 2**	**Week 4**	**Week 8**
Inclusion/Exclusion criteria	**×**			
Informed consent	**×**			
History/Demographics	**×**			
RPFS	**×**	**×**	**×**	**×**
ECOG-PS	**×**	**×**	**×**	**×**
SRSS	**×**	**×**	**×**	**×**
HADS	**×**	**×**	**×**	**×**
Safety assessment	**×**		**×**	
Adverse events		**×**	**×**	**×**
Combined medication		**×**	**×**	**×**

*Abbreviations*: ECOG-PS Eastern Cooperative Oncology Group Performance Status; HADS, Hospital Anxiety and Depression Scale; RPFS, Revised Piper Fatigue Scale; SRSS, Self-Rating Scale of Sleep.

### Serious adverse event reporting and monitoring

Any serious adverse events deemed to be related to the intervention or due to study participation will be reported to the chief investigator within 24 hours. Beijing Qihuang Medicine Clinical Research Center will be responsible for quality control.

## Discussion

CRF is a significant problem in as many as 40% of disease-free patients and impacts their quality of life. The National Comprehensive Cancer Network provides an evidence-based guideline that include pharmacological and non-pharmacological interventions [2]. However, it does not include TCM, which effectively relieves the symptoms of CRF. This trial will explore its effectiveness as an intervention to support patients with breast cancer and CRF. To our knowledge, this will be the first Chinese herbal formula intervention designed to reduce CRF. This study has the potential to contribute to the development of an effective intervention to help relieve CRF.

Our study also has several limitations that require consideration. First, we did not design subjective measures to assess CRF changes. Metrics for both subjective and objective measures, including inflammatory and genetic markers, are recommended [1]. Second, the primary outcome RPFS is not often used in clinical research with breast cancer patients. Many questionnaires for CRF use a single-dimensional or multidimensional perspective [13]. However, there is no acknowledged questionnaire. We hope we can improve on these problems in future research.

### Trial status

We are currently recruiting participants.

## Abbreviations

CRF: Cancer-related fatigue
ECOG-PS: Eastern cooperative oncology group performance status
GMP: Good manufacturing practice
HADS: Hospital anxiety and depression scale
P: Placebo
RPFS: Revised piper fatigue scale
SGJP: *Shu Gan Jian Pi*
SRSS: Self-rating scale of sleep
TCM: Traditional chinese medicine
